# Supplementary material for: BLOS2 negatively regulates Notch signaling during neural and hematopoietic stem and progenitor cell development
Source: eLife. 2016 Oct 10;5:e18108. doi: 10.7554/eLife.18108 (PMC5094856; doi:10.7554/eLife.18108)
Supplement: Figure 3—source data 1. — DOI: http://dx.doi.org/10.7554/eLife.18108.007 [file elife-18108-fig3-data1.pdf]

Figure 3A-source data 1

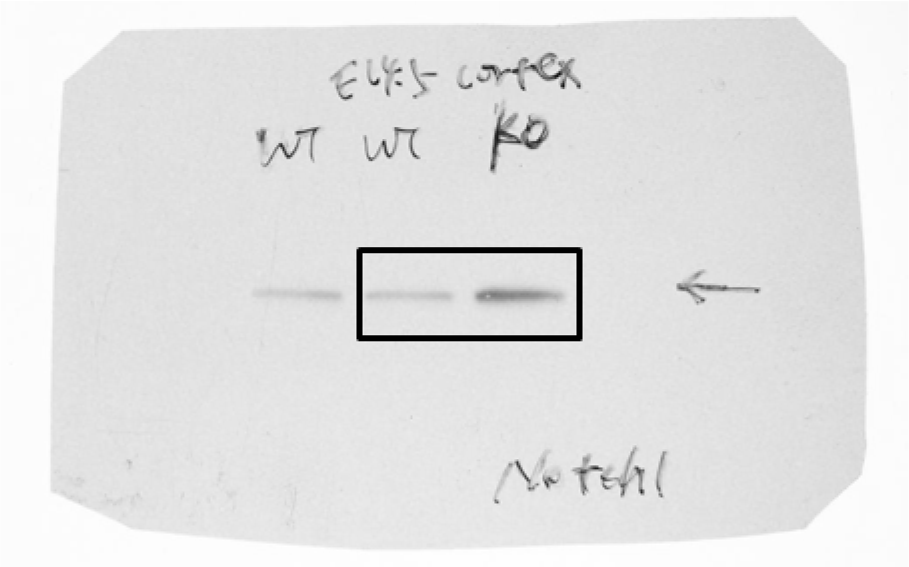

The black box shows the bands of Notch1 (N^{TM}).

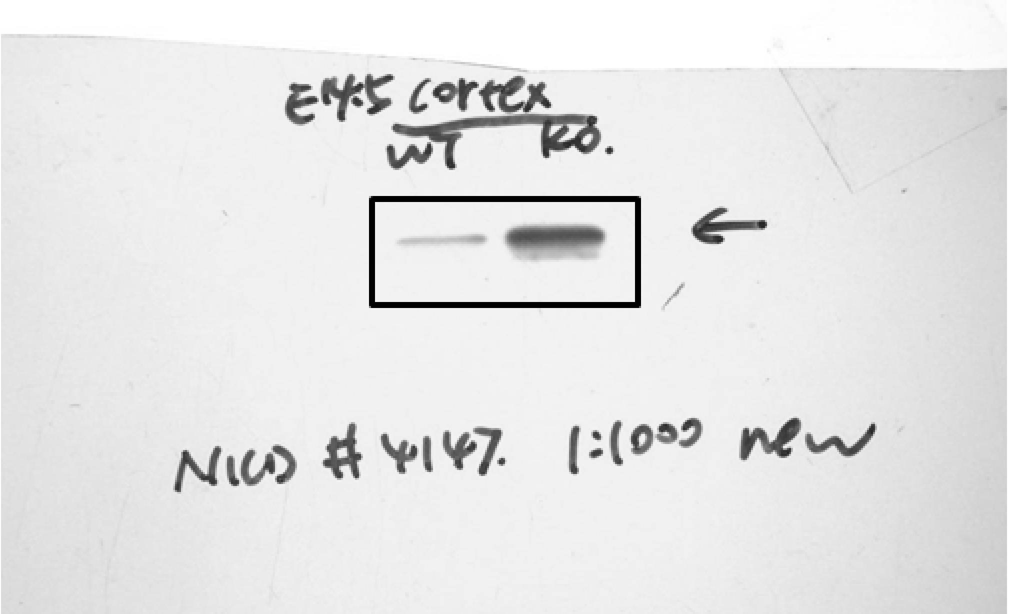

The black box shows the bands of NICD1.

Figure 3A-source data 2

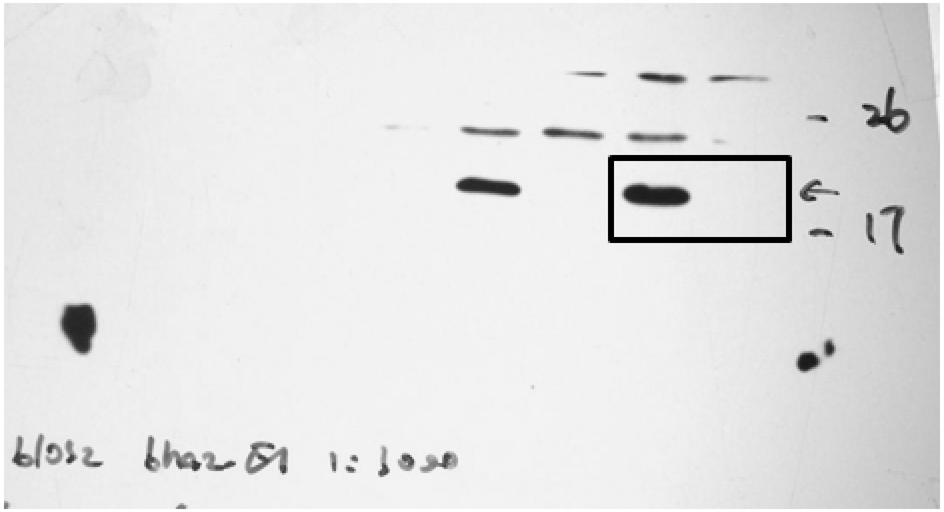

The black box shows the bands of BLOS2.

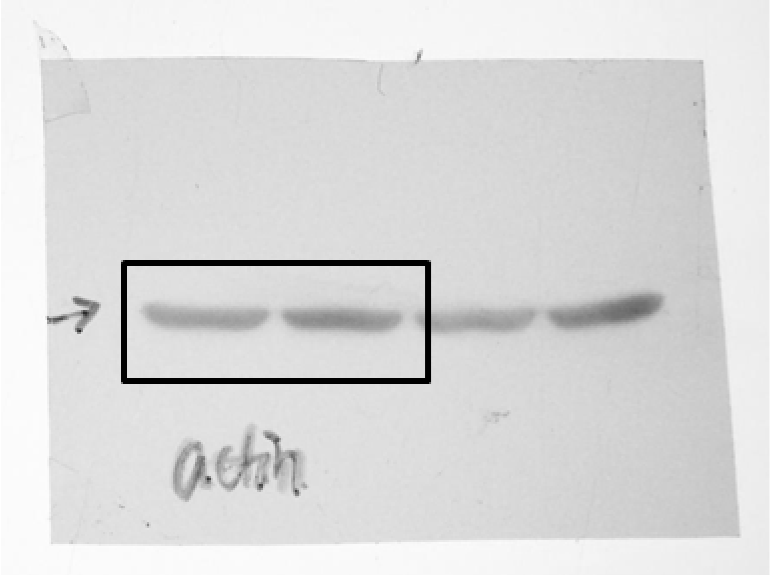

The black box shows the bands of β-actin.
