# Supplementary figures and images for: BLOS2 negatively regulates Notch signaling during neural and hematopoietic stem and progenitor cell development
Source: eLife. 2016 Oct 10;5:e18108. doi: 10.7554/eLife.18108 (PMC5094856; doi:10.7554/eLife.18108)

Figure 4A-source data 1

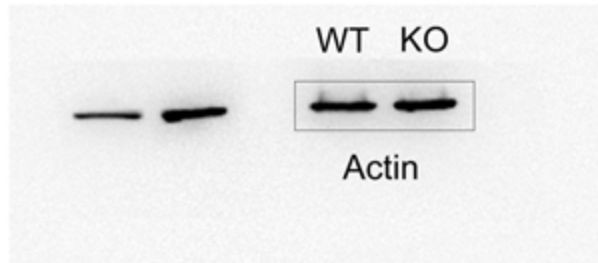

The black box shows the bands of Actin .

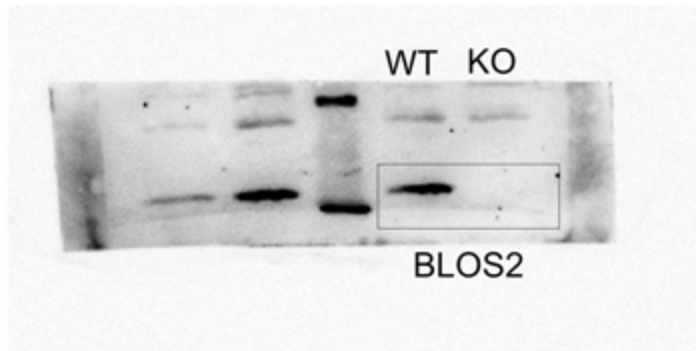

The black box shows the bands of BLOS2.

Supplement: Figure 4—source data 1. — DOI: http://dx.doi.org/10.7554/eLife.18108.016 [file elife-18108-fig4-data1.pdf]

Figure 5C-source data 1

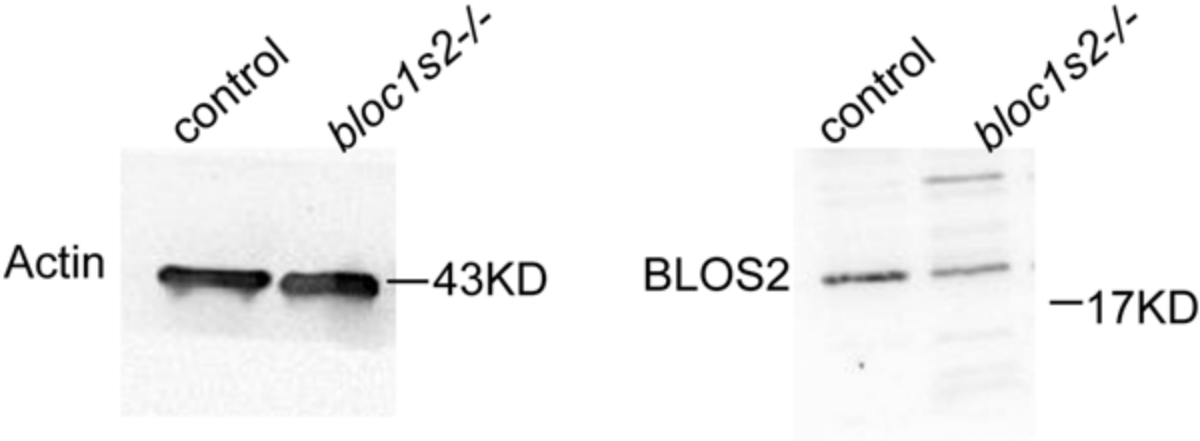

Supplement: Figure 5—source data 1. — DOI: http://dx.doi.org/10.7554/eLife.18108.020 [file elife-18108-fig5-data1.pdf]

Figure 5D-source data 1

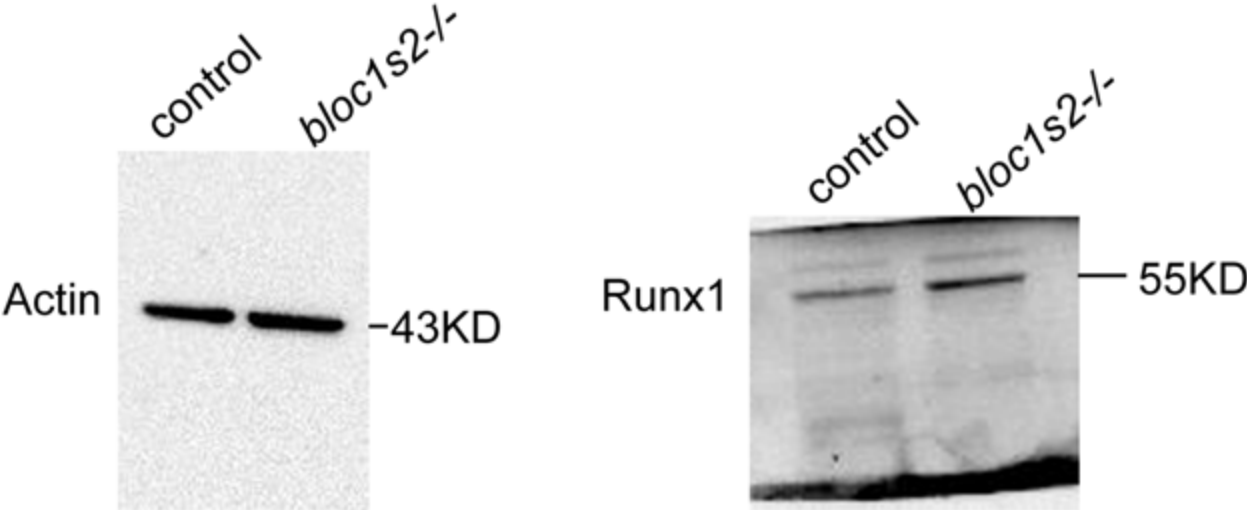

Supplement: Figure 5—source data 2. — DOI: http://dx.doi.org/10.7554/eLife.18108.021 [file elife-18108-fig5-data2.pdf]

Figure 5-figure supplement 2D-source data 1

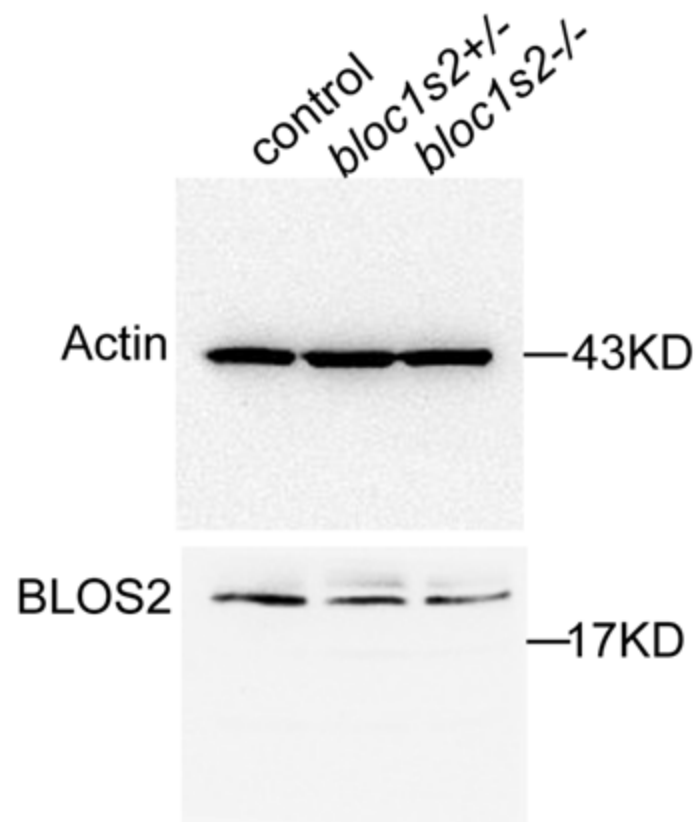

Supplement: Figure 5—figure supplement 2—source data 1. — DOI: http://dx.doi.org/10.7554/eLife.18108.024 [file elife-18108-fig5-figsupp2-data1.pdf]

Figure 5-figure supplement 3C-source data 1

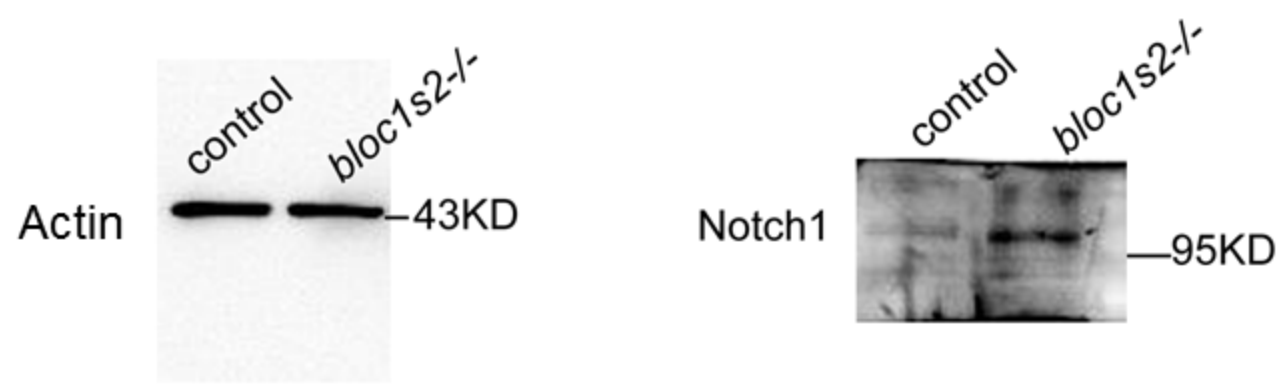

Supplement: Figure 5—figure supplement 3—source data 1. — DOI: http://dx.doi.org/10.7554/eLife.18108.026 [file elife-18108-fig5-figsupp3-data1.pdf]

Figure 6E-source data 1

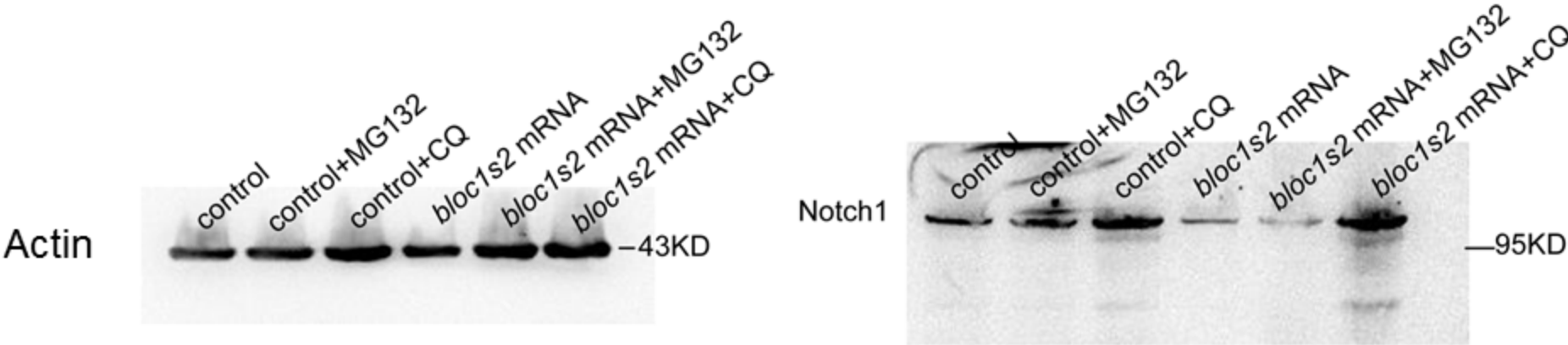

Supplement: Figure 6—source data 2. — DOI: http://dx.doi.org/10.7554/eLife.18108.029 [file elife-18108-fig6-data2.pdf]

Figure 8B-source data 1

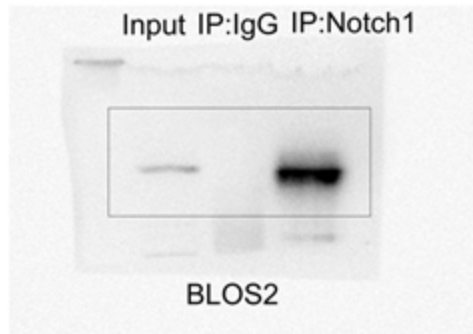

The black box shows the bands of BLOS2.

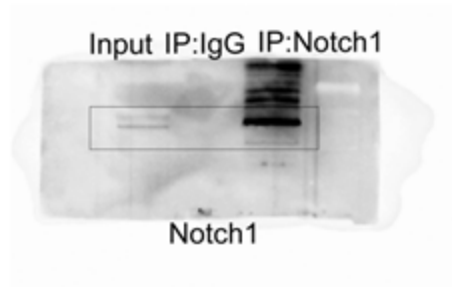

The black box shows the bands of Notch1.

Supplement: Figure 8—source data 2. — DOI: http://dx.doi.org/10.7554/eLife.18108.035 [file elife-18108-fig8-data2.pdf]
