# Supplementary material for: BLOS2 negatively regulates Notch signaling during neural and hematopoietic stem and progenitor cell development
Source: eLife. 2016 Oct 10;5:e18108. doi: 10.7554/eLife.18108 (PMC5094856; doi:10.7554/eLife.18108)
Supplement: Figure 4—source data 2. — DOI: http://dx.doi.org/10.7554/eLife.18108.017 [file elife-18108-fig4-data2.pdf]

Figure 4G-source data 1

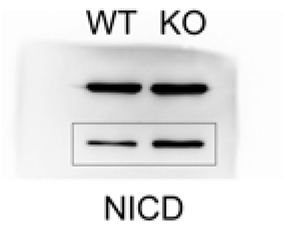

The black box shows the bands of NICD.

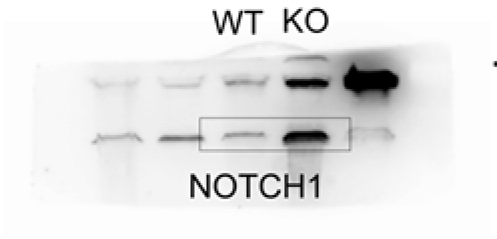

The black box shows the bands of Notch1.

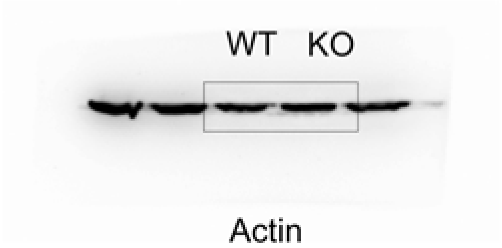

The black box shows the bands of Actin.
