# Supplementary material for: BLOS2 negatively regulates Notch signaling during neural and hematopoietic stem and progenitor cell development
Source: eLife. 2016 Oct 10;5:e18108. doi: 10.7554/eLife.18108 (PMC5094856; doi:10.7554/eLife.18108)
Supplement: Supplementary file 1. — DOI: http://dx.doi.org/10.7554/eLife.18108.045 [file elife-18108-supp1.doc]

**Supplementary file 1. A comparison of phenotypes for BLOS2-KO, BLOS1-KO and Snapin-KO mouse embryos or MEFs.**

|  | **BLOS2-****KO** | **BLOS1-KO** | **Snapin-KO** |
| --- | --- | --- | --- |
| Embryonic lethality | Yes | Yes | Yes |
| Small cerebral cortex | Yes | N/A | Yes |
| Increased neuron death in the cortex | No | N/A | Yes |
| Impaired hematopoiesis | Yes | N/A | N/A |
| Craniofacial malformation | Yes | No | N/A |
| Loss of eye pigmentation | Yes | Yes | N/A |
| Impaired autophagy | No | Yes | Yes |
| Delayed EGFR degradation | No | Yes | Yes |
| Upregulated Notch signaling | Yes | No | N/A |
| References | This study |  |  |

(N/A: not applicable.)

References:

Cai Q, Lu L, Tian JH, Zhu YB, Qiao H, Sheng ZH. 2010. Snapin-regulated late endosomal transport is critical for efficient autophagy-lysosomal function in neurons. *Neuron* **68**:73-86. doi:10.1016/j.neuron.2010.09.022.

Tian JH, Wu ZX, Unzicker M, Lu L, Cai Q, Li C, Schirra C, Matti U, Stevens D, Deng C, Rettig J, Sheng ZH. 2005. The role of Snapin in neurosecretion: snapin knock-out mice exhibit impaired calcium-dependent exocytosis of large dense-core vesicles in chromaffin cells. *J Neurosci* **25**:10546-55. doi:10.1523/JNEUROSCI.3275-05.2005.

Zhang A, He X, Zhang L, Yang L, Woodman P, Li W. 2014. Biogenesis of lysosome-related organelles complex-1 subunit 1 (BLOS1) interacts with sorting nexin 2 and the endosomal sorting complex required for transport-I (ESCRT-I) component TSG101 to mediate the sorting of epidermal growth factor receptor into endosomal compartments. *J Biol Chem* **289**:29180-94. doi:10.1074/jbc.M114.576561.
