# Supplementary material for: BLOS2 negatively regulates Notch signaling during neural and hematopoietic stem and progenitor cell development
Source: eLife. 2016 Oct 10;5:e18108. doi: 10.7554/eLife.18108 (PMC5094856; doi:10.7554/eLife.18108)
Supplement: Supplementary file 2. — DOI: http://dx.doi.org/10.7554/eLife.18108.046 [file elife-18108-supp2.doc]

**Supplementary file 2. The PCR primers used in quantitative PCR assay.**

| **gene** | | **Primer name** | **qPCR primer sequence (5’→3’)** |
| --- | --- | --- | --- |
| zebrafish | *β-actin* | *β-actin F* | GCTGTTTTCCCCTCCATTGTT |
|  |  | *β-actin R* | TCCCATGCCAACCATCACT |
|  | *runx1* | *runx1* F | ACCGTCTTCACAAACCCTC |
|  |  | *runx1* R | CCTGGCTTTACTGCTTCATC |
|  | *cmyb* | *cmyb* F | TGATGCTTCCCAACACAGAG |
|  |  | *cmyb* R | TTCAGAGGGAATCGTCTGCT |
|  | *hey2* | *hey2* F | ATTGATGTGGGCAGCGAGAA |
|  |  | *hey2* R | TGGGATGTGGTGGATGTGGA |
|  | *bloc1s2* | *bloc1s2* F | AGCCGAGCCCGAGAACA |
|  |  | *bloc1s2* R | CTCCCTGCAAGAAAACCGA |
|  | *ephrinB2a* | *ephrinB2a* F | CAAGGACAGCAAATCGAATG |
|  |  | *ephrinB2a* R | TGAGCCAATGACTGATGAGG |
| mouse | *Hey1* | *Hey1* F | GCGCGGACGAGAATGGAAA |
|  |  | *Hey1* R | TCAGGTGATCCACAGTCATCTG |
|  | *Hey2* | *Hey2* F | AAGCGCCCTTGTGAGGAAAC |
|  |  | *Hey2* R | GGTAGTTGTCGGTGAATTGGAC |
|  | *Gapdh-1* | *Gapdh* F | TCCCACTCTTCCACCTTCGATGC |
|  |  | *Gapdh* R | GGGTCTGGGATGGAAATTGTGAGG |
|  | *Hes1* | *Hes1* F | TGAAGGATTCCAAAAATAAAATTCTCTGGG |
|  |  | *Hes1* R | CGCCTCTTCTCCATGATAGGCTTTGATGAC |
|  | *Hes5* | *Hes5* F | TTCCTTTGTATGGGTGGGTGC |
|  |  | *Hes5* R | GAAGCCTTCAGA ACAGCCTGTG |
|  | *Notch1* | *Notch1* F | AGGACTGTCAGACTGTGGCTTAGC |
|  |  | *Notch1* R | ATCCTGGGTTGTGCTCTTAGGAG |
|  | *Dll1* | *Dll1* F | AAGGATATAGCCCCGATGAATGC |
|  |  | *Dll1* R | TGCTAACTCTGAGAGAACCAGCTTCG |
|  | *Itch* | *Itch* F | TCACTTGGGCATAGGTCTCT |
|  |  | *Itch* R | TGTGCCCAGACACTGAGTTA |
|  | *Dtx2* | *Dtx2* F | CCCCTTACATCATCGACCTCC |
|  |  | *Dtx2* R | GCGCACAGACCTCATGGTG |
|  | *Fabp7* | *Fabp7* F | AGTCTGTGGTTCGGTTGGAT |
|  |  | *Fabp7*R | TAACAGCGAACAGCAACGAT |
|  | *Gapdh-2* | *Gapdh-2* F | TGCGACTTCAACAGCAACTC |
|  |  | *Gapdh-2* R | CTTGCTCAGTGTCCTTGCTG |
